# Vitamin D deficiency and supplementation in patients with aggressive B‐cell lymphomas treated with immunochemotherapy

**DOI:** 10.1002/cam4.1166

**Published:** 2017-12-22

**Authors:** Stefan Hohaus, Maria Chiara Tisi, Silvia Bellesi, Elena Maiolo, Eleonora Alma, Germana Tartaglia, Francesco Corrente, Annarosa Cuccaro, Francesco D'Alo', Umberto Basile, Luigi Maria Larocca, Valerio De Stefano

**Affiliations:** ^1^ Institute of Hematology Università Cattolica del Sacro Cuore Rome Italy; ^2^ Department of Laboratory Medicine Università Cattolica del Sacro Cuore Rome Italy; ^3^ Institute of Pathological Anatomy Università Cattolica del Sacro Cuore Rome Italy

**Keywords:** Aggressive B‐cell lymphoma, prognosis, supplementation, Vitamin D

## Abstract

Vitamin D deficiency has been reported to be a negative prognostic factor in elderly patients with aggressive B‐cell lymphomas. In vitro data suggest that vitamin D supplementation may enhance rituximab‐mediated cytotoxicity. We prospectively assessed 25‐hydroxyvitamin D [25(OH)D] levels at diagnosis in a cohort of 155 patients with aggressive B‐cell lymphomas of whom 128 had diffuse large B‐cell lymphoma (DLBCL) not otherwise specified. 25(OH)D levels were deficient (<20 ng/mL) in 105 (67%), insufficient (20–29 ng/mL) in 32 (21%), and normal (≥30 ng/mL) in 18 (12%) patients with a seasonal variation. Patient characteristics associated with lower 25(OH)D levels were poor performance status, overweight, B‐symptoms, elevated LDH, lower albumin and hemoglobin levels. As a result of a change in practice pattern, 116 patients received vitamin D3 (cholecalciferol) supplementation that included a loading phase with daily replacement and subsequent maintenance phase with a weekly dose of 25,000 IU until end of treatment. This resulted in a significant increase in 25(OH)D levels, with normalization in 56% of patients. We analyzed the impact of 25(OH)D levels on event‐free survival in patients treated with Rituximab‐CHOP. 25(OH)D levels below 20 ng/mL at diagnosis and IPI were independently associated with inferior EFS. Moreover, patients with normalized 25(OH)D levels following supplementation showed better EFS than patients with persistently deficient/insufficient 25(OH)D levels. Our study provides the first evidence that achievement of normal 25(OH)D levels after vitamin D3 supplementation is associated with improved outcome in patients with DLBCL and deficient/insufficient 25(OH)D levels when receiving rituximab‐based treatment.

## Introduction

Treatment results of diffuse large B‐cell lymphoma (DLBCL) have significantly improved after the introduction of immunotherapy with the monoclonal CD20 antibody rituximab, added to traditional chemotherapy regimens 1, 2. Recently, an association between levels of vitamin D and lymphoma mortality has been reported for patients with DLBCL 3. Data from the German RICOVER‐60 study actually indicated that vitamin D deficiency is a negative prognostic factor for elderly patients with DLBCL, treated with rituximab‐containing chemotherapy (R‐CHOP) 4. Similarly, low serum vitamin D levels were identified as negative prognostic factor for survival in a retrospective analysis of cohorts of patients with follicular lymphoma (FL) 5.

The main blood circulation form of vitamin D is 25‐hydroxyvitamin D3 [25(OH)D], and its level is a good indicator of vitamin D status. Vitamin D deficiency, defined as a serum 25(OH)D level below 10 ng/mL, is very common in the elderly population, and this is mainly explained by low dietary vitamin D intake and the lack of sufficient sunlight exposure 6. A growing body of evidence indicates that vitamin D deficiency may play a role in the development of common cancers, although a recent meta‐analysis did not find an association between risk for non‐Hodgkin lymphoma (NHL) and vitamin D deficiency 7, 8, 9.

Vitamin D deficiency is well known for its musculoskeletal complications 10. However, vitamin D receptors (VDR) are also present on a large variety of extraskeletal cell types, including cells of the immune system, where vitamin D has been shown to play a role in both innate and adaptive immune responses 11, 12. Antibody‐mediated cytotoxicity appears to be a major mechanism by which rituximab exerts its B‐cell depleting activity 13. In this context, vitamin D supplementation has been shown to enhance rituximab‐mediated cytotoxicity in vitro 14. It is unknown at present whether these mechanisms are active as well during treatment of lymphoma patients.

The Institute of Medicine (IOM) in the U.S. recommended a dietary intake of vitamin D 600–800 U/day for the general population 15. Guidelines targeting patients recommend higher supplementation doses 16. Supplementation studies are mainly limited to elderly people, where efficacy of the regimen is usually assessed after 4–6 months or longer 17. Although supplementation may help to improve vitamin D status in the long run, there is currently no consensus on how to achieve this target rapidly, especially in cases of severe vitamin D deficiency.

The time required to achieve 25(OH)D concentrations in the normal range could be a critical issue in the context of NHL treatment. We aimed at rapid normalization of vitamin D levels to increase rituximab‐efficacy early on during therapy. We therefore applied an intensive supplementation regimen with a daily loading phase of 25,000 IU vitamin D3, tailored to the degree of deficiency, followed by a maintenance phase of 25,000 IU once weekly till the end of immunotherapy. The aim of our study was to develop a practical regimen of oral substitution of vitamin D3 (cholecalciferol) that would enable rapid improvement of vitamin D deficiency, and maintenance of normal levels during treatment with rituximab‐containing regimens. A secondary objective was to analyze for clinical benefits of vitamin D replacement.

## Subjects and Methods

### Patient characteristics

We studied 155 patients (89 females, 66 males) with a median age of 65 years, diagnosed with aggressive B‐cell lymphoma and treated at the Department of Hematology of the Catholic University of the Sacred Heart, Gemelli Foundation, in Rome between September 2013 and June 2016. Histology was: DLBCL not otherwise specified (NOS) (*n* = 128), primary mediastinal large B‐cell lymphoma (*n* = 9), B‐cell lymphoma, unclassifiable‐intermediate between DLBCL and Burkitt lymphoma (*n* = 8), T‐cell/histiocyte‐rich DLBCL (*n* = 4), and other forms (*n* = 6), according to the 2008 WHO classification 18. All patients were treated with rituximab‐containing chemotherapy. The standard regimen was R‐CHOP (rituximab, cyclophosphamide, doxorubicin, vincristine, prednisolone) given for six cycles of CHOP and eight cycles of rituximab, with reduction in doxorubicin by 50% in 12 patients and omission in 26 elderly unfit patients 1, 2.

The study was approved by our institutional review board and conducted according to the Declaration of Helsinki. All patients provided written consent.

### Measurement of serum 25‐hydroxyvitamin D [25(OH)D] levels

Serum samples for vitamin D quantification were collected before the first day of immune‐chemotherapy as pretreatment control samples. Follow‐up samples were then obtained on the first visit following the 1–2 week loading phase, and at least 10 days after the final loading dose of cholecalciferol. This interval was chosen because previous studies showed that about 10 days are required to convert 90–100% of the absorbed cholecalciferol into 25(OH)D 19.

25(OH)D was assayed in the central laboratory of the Academic Hospital A. Gemelli, Rome, in a blinded fashion and in a single batch and measured in patients' sera using a standardized clinical assay, the DiaSorin LIAISON 25‐OH Vitamin D TOTAL, a fully automated sensitive immunoassay that uses a recombinant fusion construct of the vitamin D receptor ligand‐binding domain for specific capture of 25(OH)D. The dynamic range of the assay is 4.0–150 ng/mL. The lowest value in the laboratory report was <7.0 ng/mL, and for statistical purposes, values <7.0 ng/mL were scored as 7.0 ng/mL. For the purpose of analysis, 25(OH)D levels were defined according to three conditions: deficient (<20 ng/mL), insufficient (20–29 ng/mL), and normal (≥30 ng/mL) 20.

### Vitamin D supplementation

**Figure 1:**
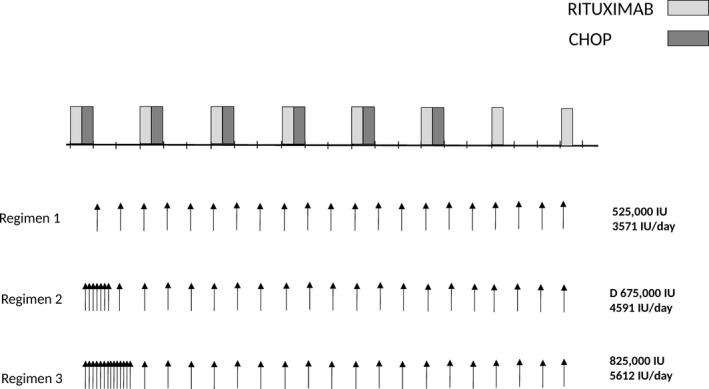
Supplementation with vitamin D3 during rituximab‐containing chemotherapy. Three different vitamin D3 (cholecalciferol) regimens were applied. Regimen 1 consisted in weekly administration of 25,000 IU vitamin D3. In regimen 2 and 3, patients started with a loading phase of vitamin D3 25,000 IU daily for 7 or 14 days, respectively, and then continued with 25,000 IU weekly till the end of therapy.

Patients with 25(OH)D levels lower than 30 ng/mL were candidates for vitamin D supplementation. As practice pattern changed over time, 27 patients with 25(OH) levels below 30 ng/mL were left without supplementation (Table S1). Cholecalciferol (vitamin D3) was used for vitamin D supplementation and was taken orally as liquid solution, poured on a piece of bread to enhance compliance. As dose guidelines for rapid loading were not available, we designed a pragmatic dose‐escalation schedule that was considered safe to conduct as part of regular patient care (Fig. 1, Table S1). To minimize the risk of oversupplementation, the first group of patients (regimen 1, *n* = 35) was treated with a weekly dose of 25,000 IU vitamin D3. Once laboratory results showed that this dose did not induce hypercalcemia, and that 25(OH)D levels had only modestly increased with rarely hitting the target level of normal (≥30 ng/mL), we added a loading phase with 25,000 IU vitamin D3 daily for 1 week. Preliminary data confirmed that patients with severe deficiency defined by very low 25(OH)D levels (<10 ng/mL) did not reach the target of normal 25(OH)D levels with the 1 week loading dose. We then increased the duration of daily vitamin D3 supplementation to 2 weeks in this patient group. Following the initial cholecalciferol load, the treatment was continued using a maintenance dose of 25,000 IU weekly, until completion of treatment, including six cycles of R‐CHOP and two additional rituximab doses at 375 mg/m^2^, for a total of 21 weeks. At each cycle, patient calcium levels were measured. These regimens correspond to a total cholecalciferol dose of 175,000 IU in the 1‐week and 350,000 units in the 2‐week schedule during the loading phase, and of 500,000 IU during the maintenance phase of eight rituximab‐CHOP cycles.

### Statistical analysis

Results are expressed as median values ± SD. As 25(OH)D levels show seasonal variations, we analyzed pretreatment samples according to the season: (1) winter: January through March (2) spring: April through June, (3) summer: July through September, and (4) fall: October through December. Wilcoxon signed‐rank test was used for two‐sample comparisons of 25(OH)D levels according to dichotomized patient characteristics, and the Kruskal–Wallis test, when there were more than two groups.

Changes in 25(OH)D levels following the loading phase were analyzed by paired, two‐sided *t*‐tests. To enable calculation procedures, 25(OH)D below the assay's detection limit was given a value of 7 ng/mL. Multivariate logistic regression analysis was used to identify the variables that might predict the response to cholecalciferol loading dose. Variables included for this analysis were: age, body mass index (BMI), supplementation regimen, and baseline serum 25(OH)D.

The primary survival end point was event‐free survival (EFS), with progression during treatment, lack of complete remission at the end of first‐line treatment, relapse and death from any cause counted as events. Survival curves were estimated using the Kaplan–Meier product limit method. Log‐rank tests were used to analyze for differences in EFS. Cox regression was used for multivariate analysis of EFS including dichotomized (25/OH)D levels and the international prognostic score (IPI) (score 0–2 vs. 3–5). A *P* < 0.05 was considered statistically significant. Computations were performed using the Stata 10.0 software (Stata Corp., College Station, TX).

## Results

### Analysis of 25(OH)D levels in patients with aggressive B‐cell lymphomas and associations with patient characteristics

**Figure 2:**
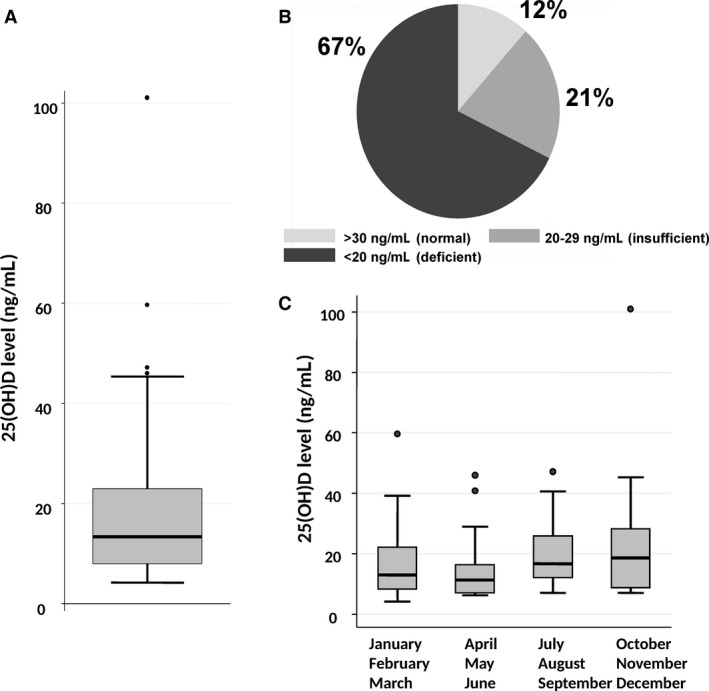
25(OH)D levels in 155 patients with aggressive B‐cell lymphomas. (A) Box‐plot of 25(OH)D levels. The upper border of the box indicates the upper quartile (75th percentile) while the lower border indicates the lower quartile (25th percentile), and the horizontal line in the box the median. The vertical lines are the whiskers indicating the upper and lower adjacent values. (B) Distribution of patients according to 25(OH)D levels considered to be normal (≥30 ng/mL), insufficient (20–29 ng/mL), or deficient (<20 ng/mL). C) Seasonal variation in 25(OH)D levels were significantly lower in the period from April to June (*P* < 0.001).

The median 25(OH)D level at diagnosis, before start of therapy, was 14 ng/mL (range: <7–101 ng/mL) in 155 patients with aggressive B‐cell lymphomas (Fig. 2A). 25(OH)D levels were deficient (<20 ng/mL) in 105 patients (67%), insufficient (20–29 ng/mL) in 32 patients (55%), and normal (≥30–100 ng/mL) in 18 patients (12%) (Fig. 2B).

Looking at patient characteristics, 25(OH)D levels were lower in patients with reduced performance status (ECOG ≥ 2) (*P* = 0.002), overweight (body mass index > 25 kg/m^2^), *P* = 0.05], presence of B‐symptoms (*P* = 0.05), albumin <4 g/L (*P* = 0.003), hemoglobin below 10 g/dL (*P* = 0.01), and elevated LDH (*P* = 0.0007) (Table 1). In addition, there was a significant seasonal variation, with lowest 25(OH)D levels after winter, and highest levels after summer (*P* = 0.009) (Table 1, Fig. 2C).

**Table 1:** Patient Characteristics and 25(OH)D levels

Variables	Patients (n)	25(OH)D, ng/mL, (median)	*P*
Total	155	14	
Histology			
NOS	128	15	0.5
PBML	9	15	
TCHR	4	17	
Unclassifiable	8	14	
Other	6	14	
Age			
<60 years	60	14	0.8
>60 years	95	15	
Gender			
Female	89	13	0.5
Male	66	15	
Body mass index			
<25 kg/m^2^	90	16	**0.05**
>25 kg/m^2^	64	11	
Stage			
I–II	58	14	0.5
III–IV	97	14	
B‐symptoms			
No	108	15	**0.05**
Yes	44	9	
ECOG			
<2	118	16	**0.002**
≥2	37	10	
IPI risk group			
Low (0‐1)	40	17	0.1
Intermediate (2‐3)	110	13	
High (4‐5)	5	14	
IPI age‐adjusted			
Low (0–1)	78	15	0.1
High (2–3)	77	13	
LDH			
Normal	72	17	**0.0007**
Elevated	83	12	
Albumin, g/dL			
≥4	68	18	**0.003**
<4	87	12	
Hemoglobin, g/dL			
≥10	129	15	**0.01**
<10	26	9	
Time period			
January–March	56	13	**0.01**
April–June	36	11	
July–September	36	17	
October–December	27	19	

NOS indicates not otherwise specified; PBML, primary mediastinal large B‐cell lymphoma; TCHR, T‐cell/histiocyte‐rich large B‐cell lymphoma; Unclassifiable, lymphoma with intermediate characteristics between DLBCL and Burkitt lymphoma. Significant values are indicated in bold.

### Correction of 25(OH)D levels with different supplementation regimens

**Figure 3:**
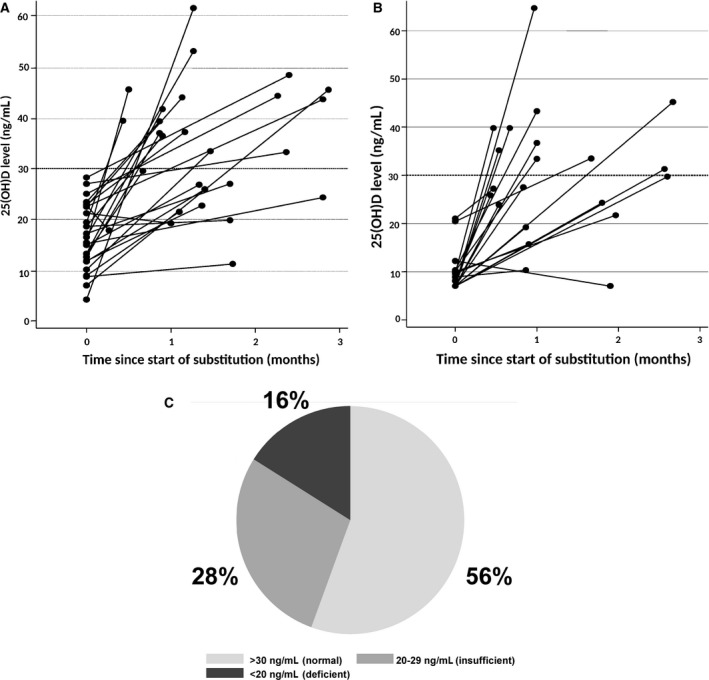
Variation in 25(OH)D levels during supplementation. (A) in patients supplemented with vitamin D3 according to regimen 1, (B) patients supplemented with regimen 2. (C) Proportion of patients with 25(OH)D levels during supplementation in the normal (≥30 ng/mL), insufficient (20–29 ng/mL), and deficient (<20 ng/mL) range. 25(OH)D levels were measured in 81 patients.

We next considered whether oral supplementation with vitamin D3 (cholecalciferol) would rapidly restore 25(OH)D levels to normal range, and changed our practice pattern to supplement vitamin D3. As a consequence of this change, a total of 116 patients received oral supplementation with cholecalciferol. A supplementation regimen of 25,000 IU once weekly was used in a group of 35 patients. The increase in 25(OH)D level was modest (median increase by 1.3‐fold after a median of 3 months, *n* = 21 patients, Table 2). We therefore designed a supplementation regimen that started with a loading phase consisting of a 1‐week course of daily vitamin D supplementation (regimen 2), that we extended to 2 weeks in patients with severely deficient levels (<10 ng/mL) (regimen 3). The loading phase was followed by a maintenance phase of once weekly vitamin D3 administration (Fig. 1). This resulted in 1.9‐ and 3.6‐fold increase in 25(OH)D levels, respectively (Table 2). As a consequence, a second determination at a median of 6 weeks after supplementation start showed a significant increase in 25(OH)D levels from 14 ± 1.4 ng/mL to 33 ± 1.4 in 81 patients for whom 25(OH)D levels were available at a median of 6 weeks following start of supplementation (mean ± SEM, *P* < 0.0001). (Fig. 3A and B; Table 2). No episodes of hypervitaminosis or hypercalcemia were observed. Supplementation resulted in normalization of 25(OH)D levels in 45/81 patients (56%) (Fig. 3C). In 16 patients without supplementation, 25(OH)D levels showed no significant variation over time (Table 2), while the two loading regimens induced normalization of 25(OH)D levels (>30 ng/mL) in 20/34 (59%) patients with insufficient/deficient levels (regimen 2) and in 13/26 patients (50%) with severely deficient levels (regimen 3).

**Table 2:** Variation in 25(OH)D levels during therapy

Regimen^a^	Number of Patients^b^	25(OH)D at diagnosis, ng/mL (mean)	25(OH)D during therapy ng/mL (mean)	Number of Patients with normal 25(OH)D at diagnosis	Number of Patients with normal 25(OH)D during therapy	Fold Difference vs. baseline (median)	Fold Difference vs. baseline (95% CI)	*P* c
0	16/39	23.0	19.4	6/16 (%) (38%)	3/16 (19%)	1.10	0.57‐1.38	0.3
1	21/35	25.6	34.8	6/21 (29%)	12/21 (57%)	1.31	0.99‐2.40	**0.05**
2	34/52	16.8	35.1	0/34 (0%)	20/34 (59%)	1.94	1.71‐2.22	**<0.0001**
3	26/29	9.5	30.9	0/26 (0%)	13/26 (50%)	3.59	2.54‐4.23	**<0.0001**

a Regimen 0: no vitamin D supplementation; regimen 1: Vitamin D 25,000 IU once weekly; regimen 2: Vitamin D 25,000 IU daily for 1 week, followed by vitamin D 25,000 IU once weekly; regimen 3: Vitamin D 25,000 IU daily for 2 weeks followed by vitamin D 25,000 IU once weekly.

b Number of patients with a second Vitamin D level during therapy/total number of patients in the group of regimen of supplementation.

c Comparison of 25(OH)D levels at start and during therapy using paired *t*‐test. Significant p‐values (<0.05) are indicated in bold.

We compared the supplemented dose of vitamin D3 to the calculated need, using the formula of Singh et al.^21^, that considers age, body mass index, and albumin levels in addition to the baseline 25(OH)D level. The median vitamin D need was 642,831 IU for patients with insufficient levels, and 937,713 IU for patients with deficient levels. This corresponds to 675,000 IU vitamin D3 supplemented in regimen 2 and 825,000 IU supplemented in regimen 3. Multivariate regression analysis including age, albumin levels, body mass index, baseline 25(OH)D levels, and supplementation regimen showed that only body mass index was a risk factor for not achieving normal 25(OH)D levels (OR: 1.10, 95% C.I., 1.00–1.24, *P* < 0.05).

### Associations of 25(OH)D levels and vitamin D3 supplementation with clinical outcome

Treatment response was evaluated in 142/155 patients at end of therapy. Response evaluation included PET‐CT in 137 patients. Complete remission was documented in 121 patients (78%) and partial remission in 10 patients (7%), while 11 patients (8%) were considered resistant. Response was not evaluable in 13 patients, including 8 (5%) patients who died during therapy, and 5 patients who continued treatment at other centers. 25(OH)D levels were significantly higher in patients with complete remission at end of therapy (median 16 ng/mL), when compared to patients with partial remission (median 11 ng/mL), resistant disease (median 9 ng/mL), or early death (median 8 ng/mL) (*P* = 0.03).

**Figure 4:**
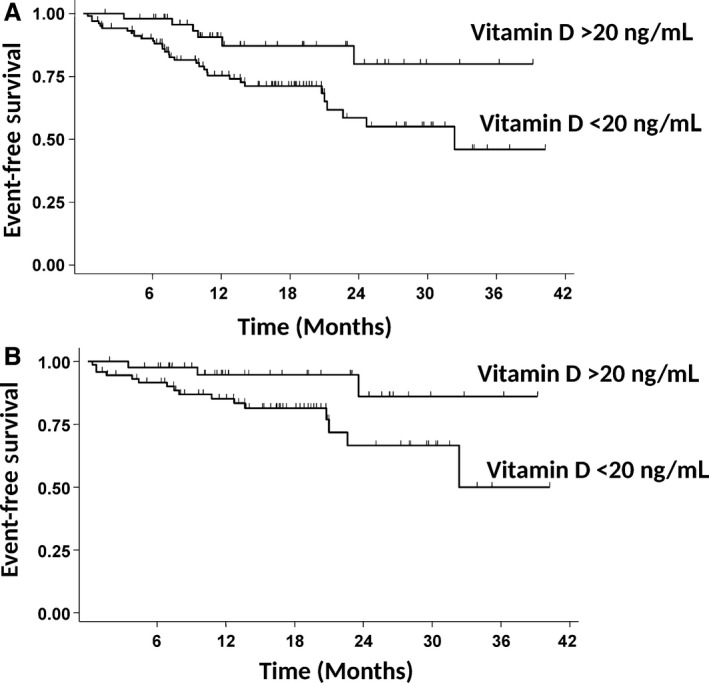
Event‐free survival in patients with aggressive B‐cell lymphoma according to 25(OH)D levels at diagnosis. (A) Group A (*n* = 50 patients) had 25(OH)D levels ≥ 20 ng/mL at diagnosis, group B (*n* = 104 patients) had 25(OH)D levels <20 ng/mL at diagnosis. The survival difference is statistically significant (*P* = 0.01). (B) The survival analysis was restricted to 116 patients treated with R‐CHOP containing full dose of doxorubicin, according to 25(OH)D levels. Group A (*n* = 43 patients) had 25(OH)D levels ≥20 ng/mL at diagnosis, group B (*n* = 73 patients) had 25(OH)D levels <20 ng/mL at diagnosis. The difference is statistically significant (*P* = 0.03).

We analyzed the prognostic impact of 25(OH)D levels at diagnosis on EFS. Testing 25(OH)D levels as a continuous variable, we found that each unit increase in 25(OH)D was associated with a reduction in the risk for event (odds ratio 0.959; 95% CI, 0.922–0.998, *P* = 0.04). Using the ROC analysis, we identified the significant cut‐off point for 25(OH)D levels at 20 ng/ml. Patients with lower levels (*n* = 104) had a significantly worse EFS than patients with higher levels (*n* = 50) (*P* = 0.01) (Fig. 4A). We then adjusted the analysis for the strongest outcome predictor in aggressive B‐cell lymphomas, the international prognostic index (IPI), using multivariate Cox regression analysis. 25(OH)D levels < 20 ng/ml and IPI were independent prognostic parameters for EFS (Table 3). This difference was maintained when the analysis was restricted to 116 patients who had been treated with R‐CHOP and full dose of doxorubicin (*P* = 0.03) (Fig. 4B). 25(OH)D levels proved to be independent from IPI also in this patient subgroup (*P* = 0.04).

**Table 3:** Multivariate analyses of event‐free survival in 154 patients (Cox regression)

Variable	HR	95% C.I.	P**
25(OH)D level at diagnosis( )<20 ng/mL vs. **≥**20 ng/mL	2.88	1.20–6.90	0.02
IPI 3–5 vs. 0–2	2.97	1.47–56.00	0.002

HR, hazard ratio, C.I., confidence interval. ** P‐values <0.05 are considered significant

**Figure 5:**
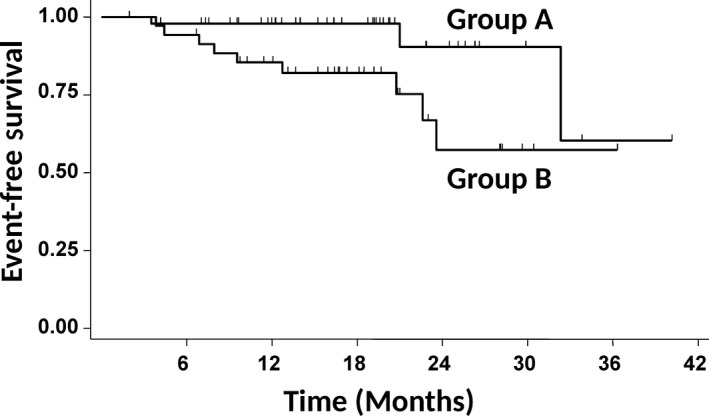
Event‐free survival in patients with aggressive B‐cell lymphoma according to 25(OH)D levels following supplementation. The survival analysis was restricted to 68 patients treated with R‐CHOP containing full dose of doxorubicin, according to 25(OH)D levels. Group A (*n* = 36 patients) had normal (≥30 ng/mL), and group B lower than normal (<30 ng/mL) 25(OH)D levels after loading phase. The survival difference is statistically significant (*P* = 0.02).

We then analyzed the prognostic impact of 25(OH)D levels following oral supplementation in patients treated with R‐CHOP and full‐dose doxorubicin. Patients with 25(OH)D levels in the normal range following supplementation (*n* = 36) had a significantly better EFS than patients with persistently lower than normal 25(OH)D levels (*n* = 32) (*P* = 0.02) (Fig. 5). Adjusting for IPI in multivariate Cox regression analysis, persistently low 25(OH)D was a risk factor for inferior EFS of borderline significance (HR 4.68; 95% C.I., 0.95–23.09; *P* = 0.06).

## Discussion

We show here that 25(OH)D levels can be rapidly and safely normalized in at least half of the patients with aggressive B‐cell lymphomas using a daily supplementation regimen with vitamin D3 (cholecalciferol). In addition, our study provides the first, though preliminary evidence that response to vitamin D supplementation may improve outcome in patients receiving conventional rituximab‐containing chemotherapy. Finally, our observations confirm that vitamin D deficiency/insufficiency is frequent in both young and elderly patients with aggressive B‐cell lymphomas, and correlate with both host‐related and disease‐related characteristics as well as with inferior outcome. We observed a seasonal variation according to sunlight exposure, with the lowest and highest levels before and after the summer, respectively.

25(OH)D levels were normal in 12% of patients in the present series. This is a considerably higher fraction than the <1% reported in the study from Bittenbring et al. in elderly patients with DLBCL from Germany 4. The median level was 14 ng/mL in our patient series. This appears to be similar to the median 25(OH)D concentration of 16 ng/mL reported for individuals who participated in the InCHIANTI study, a population‐based, prospective cohort study on aging in Tuscany, a region in central Italy 22. In a population of 294 obese individuals referred to a Metabolic Unit in Rome, only 19% had 25(OH)D levels in the normal range (>30 ng/mL) and the mean 25(OH)D level was 16 ng/mL, with significant variation between winter and summer 23. 25(OH)D levels appear to be lower in the Mediterranean area with respect to Northern Europe and North America 24. A careful case–control study matching for gender, age, season, and geographical origin will be needed to address the question, whether 25(OH)D levels in patients with DLBCL are lower than in the general population.

Associations between 25(OH)D levels and some patient characteristics are not unexpected. Poor performance status is associated with reduced physical activity and sunlight exposure, two factors that are well known to influence 25(OH)D levels 20. Associations with low albumin levels could probably indicate lower dietary vitamin D intake or reduced hydroxylation in the liver, the major site for 25(OH)D generation. Moreover, we observed associations with parameters of disease activity such as LDH and presence of B‐symptoms. The mechanism underlying this association is unclear. B‐symptoms are the clinical sign of inflammatory cytokine production by the tumor. In a population study, lower 25(OH) D levels have been associated with higher levels of IL‐6 25. In the same line, the inverse association between 25(OH)D and hemoglobin is not unexpected. Recent data indicate that expression of the iron‐regulatory peptide hepcidin can be modulated by vitamin D, pointing to a role for vitamin D in the development of anemia of chronic inflammation 26, 27, 28. We have previously shown that the IL‐6/hepcidin axis is frequently active in patients with Hodgkin and aggressive B‐cell lymphomas 29, 30.

We confirm here the prognostic value of baseline 25(OH)D levels on outcome, that has been previously reported in elderly patients with aggressive B‐cell lymphoma 4. In a retrospective analysis, we determined that patients with 25(OH)D levels below 20 ng/mL had a significantly lower EFS. In the German study, the threshold value was 8 ng/mL, while in the study on follicular lymphoma, the prognostically relevant threshold varied between patients from France (10 ng/mL) and the United States (20 ng/mL) 5. Further studies are needed to clarify whether these variable thresholds reflect geographical or ethnic differences, and to elucidate the role of other factors such as climate, diet, or other lifestyle habits. The limited number of patients in our study did not allow for multiple adjustments of the survival analysis for the confounding patient characteristics. We therefore cannot exclude that 25(OH)D level is a surrogate marker for the health status of the patient.

As there is a good biological rationale for the role of vitamin D in improving the antibody‐dependent antitumor macrophage activity 14, we supplemented vitamin D in patients with low 25(OH)D levels. We chose cholecalciferol (vitamin D3), as this form appears to be more effective than vitamin D2 18, 31. We developed a vitamin D supplementation regimen, including a loading phase according to the baseline serum 25(OH)D level. Our schedules appear to be safe as we did not observe toxic effects or hypercalcemia, and 25(OH)D levels never exceeded the normal range, although the vitamin D3 dose during maintenance corresponded to 3570 IU/day, which is higher than the recommended dose of up to 2000 IU/day. However, a limit of 10,000 IU/day has been proposed as the no adverse effect limit in healthy subjects 32 and high vitamin D3 daily doses up to 10,000 IU for 4 months were reported to be safe in patients with breast cancer 33. Moreover, vitamin D toxicity has been reported to start with chronic consumption of approximately 40,000 IU/day 34. The introduction of an initial loading phase resulted in a more significant increase in 25(OH)D levels after a median of 6 weeks compared to the weekly supplementation regimen. Nevertheless, still nearly half of the patients continued to have lower than normal 25(OH)D levels. This may suggest that patients undergoing immunochemotherapy, including corticosteroids, may be in need of higher vitamin D3 intake 35. In the same line with our results, 44% of patients with lung cancer who received a daily loading regimen with 20,000 IU vitamin D3 failed to achieve the target 25(OH)D level at an early control at 3 weeks 36. Very recently, an experience with weekly supplementation with 50,000 IU vitamin D3 in 71 lymphoma patients with vitamin D insufficiency defined as 25(OH)D levels <25 ng/mL has been reported 37. At 12 weeks all but one patient achieved 25(OH)D levels >25 ng/mL. At difference to our study, the second determination of the 25(OH)D level was performed later, when patients had already received a higher amount of vitamin D3 (600.000 IU).

We do not have a direct method to measure compliance. However, the second determination of 25(OH)D levels during supplementation might serve as an indirect indicator of compliance. As patients without supplementation did not show any significant change or a tendency for a decrease in the 25(OH)D level, and patients with regimen 2 and 3 generally showed significant increases in 25(OH)D levels (Table 2), we suspect that 4/60 (7%) patients in whom regimen 2 or 3 was prescribed and who showed a decrease and another 6/60 (10%) patients who showed only a slight increase that remained in the 95% CI of change in the second 25(OH)D determination of patients who did not receive substitution might not have been fully compliant. We estimate compliance to be around 83–93%.

The most significant factor predicting normalization of 25(OH)D levels was BMI. Adjustment of vitamin D3 dosing according to the BMI, by increasing the dose for patients with higher BMI, might be a strategy to normalize vitamin D in a greater proportion of patients 38. An association between BMI and 25(OH)D levels has been reported in a small series of lymphoma patients 39. The higher need in patients with increased BMI is due to the fat‐soluble nature of vitamin D and sequestration into adipose tissue. Higher single doses during the loading phase may be needed in these patients. Single doses of up to 200,000 IU have been reported to be safe and effective 40. However, there is evidence that very high doses may saturate hydroxylation reactions, thereby reducing the efficiency to generate 25(OH)D within a given period of time 41.

To address the question whether vitamin D3 supplementation resulted in clinical benefit, we analyzed event‐free survival according to the 25(OH)D level that had been achieved by supplementation. Patients with normal 25(OH)D levels after supplementation had a significant better event‐free survival when compared to patients with persistently low 25(OH)D levels, independently of the IPI score. To the best of our knowledge, this is the first study showing that response to vitamin D supplementation is associated with improved outcome in patients with DLBCL treated with immunochemotherapy. We cannot exclude that factors that determine the patient's ability to normalize 25(OH)D levels are the underlying mechanism for this association. Further studies, and ideally a randomized study on supplementation would be needed to confirm our observations. However, a randomized study might be difficult to perform, as detection of vitamin D deficiency is per se an indication to intervene to correct the deficiency.

Our supplementation led to 25(OH)D levels in the lower normal or insufficient range, and levels exceeded 60 ng/mL only in 5/81 (6%) patients. Serum concentrations that are considered optimal vary according to the effect of vitamin D on various tissues. Target 25(OH)D levels for optimal calcium absorption are lower when compared to other health outcomes 15, 25, 42. Parathyroid hormone (PTH) levels increase with vitamin D deficiency and might be an important indicator for the bone remodeling. It is not clear whether PTH levels might be also a surrogate marker for other functional consequences of vitamin D deficiency. The optimal 25(OH)D range for the stimulatory effect on the immune system has not been defined. We therefore do not know whether more intense supplementation resulting in higher 25(OH)D levels might increase the clinical benefit. Future studies are needed to determine the target 25(OH)D concentration necessary to optimize the biological effects of the antitumor macrophage activity.

We stopped vitamin D replacement at end of immunotherapy. It is unclear whether prolongation of vitamin D supplementation beyond anti‐lymphoma therapy could have an additional benefit on survival. Another area of interest for future studies will be whether genetic polymorphism in genes coding for proteins involved in the vitamin D pathway will modulate response to supplementation. These proteins include the vitamin D‐binding protein (DBP), that facilitates the transport of vitamin D metabolites in blood, the vitamin D receptor (VDR), a ligand‐dependent transcription factor, and enzymes of the cytochrome family that activate and inactivate vitamin D 43. Many of the genes involved in the vitamin D pathway are highly polymorphic. In particular, more than 60 polymorphism have been described for the VDR gene 44. Polymorphism has been associated with plasma levels, bioavailability, and function of vitamin D 43.

In conclusion, our study provides further evidence that vitamin D is a prognostically relevant and easily modifiable biomarker in aggressive B‐cell NHL. Further studies are needed to confirm our observations on the safety of vitamin D supplementation and its impact on treatment outcome.

## Conflicts of Interest

The authors declare no conflict of interests.

## Supporting information


**Table S1.** Supplementation regimens and 25(OH)D levels.
