# Supplementary material for: Vitamin D deficiency and supplementation in patients with aggressive B‐cell lymphomas treated with immunochemotherapy
Source: Cancer Med. 2017 Dec 22;7(1):270–81. doi: 10.1002/cam4.1166 (PMC5773978; doi:10.1002/cam4.1166)
Supplement: Supplementary file 1 — Table S1. Supplementation regimens and 25(OH)D levels. [file CAM4-7-270-s001.docx]

**Supplementary Table 1: Supplementation regimens and 25(OH)D levels**

|  | *Total number of patients* | *Normal*  *(>30 ng/ml), n* | *Insufficient*  *(10-29 ng/ml), n* | *Deficient*  *(<10 ng/ml), n* |
| --- | --- | --- | --- | --- |
| No supplementation | 39 | 12 | 14 | 13 |
| Regimen 1 | 35 | 6 | 18 | 11 |
| Regimen 2 | 52 | 0 | 45 | 7 |
| Regimen 3 | 29 | 0 | 8 | 21 |

Regimen 1: Vitamin D3 25,000 U once weekly;

Regimen 2: Vitamin D3 25,000 U daily for 1 week, followed by vitamin D3 25,000 U once weekly;

Regimen 3: Vitamin D3 25,000 U daily for 2 weeks followed by vitamin D3 once weekly
